# Toward Predicting Human Performance Outcomes From Wearable Technologies: A Computational Modeling Approach

**DOI:** 10.3389/fphys.2021.738973

**Published:** 2021-09-09

**Authors:** Tad T. Brunyé, Kenny Yau, Kana Okano, Grace Elliott, Sara Olenich, Grace E. Giles, Ester Navarro, Seth Elkin-Frankston, Alexander L. Young, Eric L. Miller

**Affiliations:** ^1^Cognitive Science Team, US Army DEVCOM Soldier Center, Natick, MA, United States; ^2^Center for Applied Brain and Cognitive Sciences, Tufts University, Medford, MA, United States; ^3^Department of Statistics, Harvard University, Cambridge, MA, United States; ^4^Department of Electrical and Computer Engineering, Tufts University, Medford, MA, United States

**Keywords:** machine learning, adaptive LASSO, human performance, stress, sleep, exercise, modeling

## Abstract

Wearable technologies for measuring digital and chemical physiology are pervading the consumer market and hold potential to reliably classify states of relevance to human performance including stress, sleep deprivation, and physical exertion. The ability to efficiently and accurately classify physiological states based on wearable devices is improving. However, the inherent variability of human behavior within and across individuals makes it challenging to predict how identified states influence human performance outcomes of relevance to military operations and other high-stakes domains. We describe a computational modeling approach to address this challenge, seeking to translate user states obtained from a variety of sources including wearable devices into relevant and actionable insights across the cognitive and physical domains. Three status predictors were considered: stress level, sleep status, and extent of physical exertion; these independent variables were used to predict three human performance outcomes: reaction time, executive function, and perceptuo-motor control. The approach provides a complete, conditional probabilistic model of the performance variables given the status predictors. Construction of the model leverages diverse raw data sources to estimate marginal probability density functions for each of six independent and dependent variables of interest using parametric modeling and maximum likelihood estimation. The joint distributions among variables were optimized using an adaptive LASSO approach based on the strength and directionality of conditional relationships (effect sizes) derived from meta-analyses of extant research. The model optimization process converged on solutions that maintain the integrity of the original marginal distributions and the directionality and robustness of conditional relationships. The modeling framework described provides a flexible and extensible solution for human performance prediction, affording efficient expansion with additional independent and dependent variables of interest, ingestion of new raw data, and extension to two- and three-way interactions among independent variables. Continuing work includes model expansion to multiple independent and dependent variables, real-time model stimulation by wearable devices, individualized and small-group prediction, and laboratory and field validation.

## Introduction

Advances in wearable sensing afford real-time non-invasive monitoring of digital and chemical physiology, behavior, and biomechanics in ambulatory individuals (Windmiller and Wang, 2013; Lou et al., 2018, 2020). Wearable consumer and medical devices can collect real-time data from diverse locations on the body (e.g., wrist, finger, chest, head, and oral cavity) and process and classify data to predict user status (Nag et al., 2017; Tseng et al., 2018; Terse-Thakoor et al., 2020). Classification can occur across one or more dimensions including stress, fatigue, sleep status, thermal load, hydration, blood glucose and oxygenation, and physical exertion (Yoo and Lee, 2010; Coppedè et al., 2014; Mokaya et al., 2016; Kutilek et al., 2017; Dunican et al., 2018; Ahn et al., 2019). In military or other high-stakes contexts, real-time readings of these outputs can provide trainers and leaders with information regarding the current physiological and behavioral status of individuals and teams (Kutilek et al., 2017; Friedl, 2018; Scheit, 2021).

Awareness of individual or group status in a given domain (e.g., heart rate) is not always sufficient for informing predictions of human performance outcomes, making it challenging to bridge the gap between state classification, what the status predicts for cognitive and physical performance, and what can be done to mitigate any predicted performance degradation (Danah and Crawford, 2012; Nafus, 2014; National Research Council, 2015; Howell et al., 2018; Parsons et al., 2019). For example, while actigraphy-based classification of sleep states might suggest low sleep quality or quantity, leaders may not understand how such states influence performance outcomes and therefore how to manage or mitigate the situation. Indeed, the relationship between sleep loss and performance outcomes is highly complex (Killgore, 2010). Similarly, saliva-based biomarkers of cortisol response via real-time tooth-borne sensing might suggest a high stress state (Singh et al., 2014; Tseng et al., 2018; Upasham et al., 2018), but the precise relationship between increased stress and performance outcomes remains elusive (Sandi, 2013).

The apparent disconnect between sensor outputs, status, and performance outcomes exists for at least three reasons. First, human cognitive and physical performance shows high variability within and across individuals, making performance challenging to reliably capture, model and predict, ultimately limiting the reliability of model outcomes (Smith et al., 2014). Second, computational modeling in this domain tends to be restricted to relatively few predictors and outcomes of interest and is computed using data from one or only a small handful of studies, ultimately limiting the applicability of model outcomes (Scheinost et al., 2019). Third, most research examining links between user states and cognitive and physical behavior is conducted in laboratory settings and may bear little resemblance to the tasks, environments, and behaviors characterizing relatively real-world settings, ultimately limiting the generalizability of model outcomes (Swets and Bjork, 1990; Terrin et al., 2003; Vergouwe et al., 2010).

To motivate our computational modeling approach to the challenge of human performance prediction, we first describe research on our three predictors of interest: stress, sleep, and physical exertion. Next, we describe research on our three outcomes of interest: reaction time, executive function, and perceptuo-motor control. We then describe our computational modeling approach, its outcomes, and its strengths and weaknesses. Finally, we discuss how our model framework can be extended to more complex prediction challenges, integrated with real-time state classification tools, and validated in laboratory and field research settings.

## Predictors of Interest: Stress, Sleep, and Physical Exertion

We chose three predictors of interest based on emerging wearable technologies and their ability to classify stress states, sleep status, and extent of physical exertion. For example, tooth-borne biosensors can monitor saliva in the oral cavity to measure alpha amylase and estimate stress states (Robles et al., 2011; Tseng et al., 2018), wrist-worn accelerometers can monitor actigraphy and estimate sleep/wake cycles, including sleep loss and deprivation (Dunican et al., 2018), and arm- or leg-worn mechanomyography and electromyography sensors can be used to characterize the intensity and duration of physical exertion (Esposito et al., 1998; Woodward et al., 2019). Given the increasing availability of these sensors, these three specific predictors are likely to be used in real-world situations.

Stress is a normal response to physiologically and emotionally challenging experiences, triggered by situations that are inherently novel, uncontrollable, socially threatening, or uncertain (Gagnon and Wagner, 2016). The brain is a critical component of a stress response in two ways; first, because it is the source of the stress response itself (i.e., the organ responsible for determining whether an experience is threatening), and second, because it is also responsible for activating physiological and behavioral responses to stress (McEwen, 2007). At a basic physiological level, stress activates the autonomic nervous system and hypothalamic-pituitary-adrenal (HPA) axis, and carries diverse neurotransmitter, hormonal, genomic, and immune implications (Axelrod and Reisine, 1984; Padgett and Glaser, 2003; Charmandari et al., 2005; McEwen, 2007; Gagnon and Wagner, 2016; Buchheim et al., 2019; Angelova et al., 2021). When an individual is exposed to a stressor, two stress systems are activated: a rapid catecholamine response, and a slower HPA axis and genomic response. The rapid catecholamine response is associated with increased epinephrine and norepinephrine release (Schwabe et al., 2012), and the slower HPA response is associated with increased glucocorticoid release that induces both non-genomic and genomic effects on the central nervous system (Sapolsky et al., 2000). The effects of these stress responses are most pronounced on brain regions with high catecholamine and glucocorticoid receptor densities, including the amygdala, striatum, hippocampus, and prefrontal cortex (Arnsten, 1998, 2009; Roozendaal et al., 2009; Kim et al., 2015). Given the diverse perceptual, cognitive, and affective functions of these brain areas, stress correspondingly carries diverse implications for sustaining mental performance. For example, research shows that stress can positively influence performance on relatively simple or well-rehearsed tasks (Broadbent, 1971; Arnsten, 2009) that do not demand prefrontal cortical engagement, but can negatively influence performance on relatively difficult tasks involving high levels of executive function (Arnsten, 1998, 2009; Cerqueira et al., 2007), which can be especially pronounced, for example during unpredictable and ambiguous military operations (Gaillard et al., 2006).

Sleep plays a critical role in sustaining life and health, with acutely or chronically disrupted sleep associated with diverse effects on brain function and systemic physiology, including hormonal, cardiovascular, immune, and metabolic effects (Medic et al., 2017). Unfortunately, most American adults report trouble falling asleep, poor quality sleep, or difficulty staying asleep (Knutson et al., 2017). Military work schedules exacerbate this issue, with chronic demands for continuous operations minimizing the quantity and quality of sleep (Mysliwiec et al., 2013). These include 24-hour work cycles, time zone changes, inconsistent lighting exposure, and night shifts, which can cause recurrent partial sleep deprivation and/or acute total sleep deprivation (Spiegel et al., 2016). Due to these challenging demands, one study demonstrated that 72% of service members reported six or fewer hours of nightly sleep, 43% reported five or fewer hours, and 18% reported four or fewer hours (Luxton et al., 2011). These statistics can be compounded by clinical disorders such as insomnia, post-traumatic stress disorder, and mild traumatic brain injury (mTBI), and carry important implications for cognitive and physical performance (Good et al., 2020). Restricted sleep quantity and quality influence blood glucose and glucose tolerance, limit immune function, increase blood levels of catecholamines and cortisol, and attenuate brain functional connectivity in regions known to modulate attention (Dinges et al., 1995; Spiegel et al., 1999; Chee and Tan, 2010; Lim et al., 2010; Besedovsky et al., 2012). While some debate exists regarding the robustness and reliability of sleep restriction or deprivation effects on various aspects of cognitive performance, it is generally accepted that it negatively influences vigilance and sustained attention (i.e., the ability to attend and respond to stimuli; Lim and Dinges, 2008), and induces high performance variability (Doran et al., 2001).

Physical exertion describes sustained physical activity at a moderate to high intensity level. According to the American College of Sports Medicine (ACSM) and Centers for Disease Control and Prevention (CDC), physical activity can be generally classified by the rate of energy expenditure: low, moderate, and vigorous (Ainsworth et al., 1993, 2000, 2011). One method for measuring rate of energy expenditure over time is through metabolic equivalents (METs), which are calculated as working metabolic rate relative to resting metabolic rate (Byrne et al., 2005). As METs increase over extended durations, an increasingly diverse set of metabolic, hormonal, and neurotransmitter effects occurs, ultimately resulting in fatigue and exhaustion. The cognitive responses that occur during or following an acute bout of physical exertion have been attributed to a wide range of physiological responses including cardiac output, reticular activation, catecholamine and glucocorticoid levels, cerebral blood oxygenation, and brain-derived neurotrophic factor (Chang et al., 2012). While many studies have been reviewed and subjected to meta-analysis to describe cognitive effects after exercise (Etnier et al., 1997; Sibley and Etnier, 2003; Lambourne and Tomporowski, 2010), relatively few have considered these effects during exercise (Chang et al., 2012). During moderate or high levels of physical exertion, studies suggest that cognitive performance is weakly but negatively affected, particularly for perceptual and processing speed tasks (Lambourne and Tomporowski, 2010). A more recent and comprehensive review and meta-analysis suggested a much more complex relationship between exertion and cognitive performance, with certain tasks being negatively and others positively related to exertion, further influenced by exercise intensity (Chang et al., 2012).

## Outcomes of Interest: Reaction Time, Executive Function, and Perceptuo-Motor Control

Cognitive processes are generally divided into two levels: low-level and higher-order (Miller and Cohen, 2001; Miller and Wallis, 2009). First, relatively low-level processes involve basic sensory, motor, and memory demands, and relatively routine habits and skills. Second, relatively higher-order processes involve more specialized executive functions that enable the control of attention, maintenance of task- and goal-related information, inhibition, and flexible and creative thought. We selected three outcomes of interest that represent both low-level and higher-order processes relevant to sustained performance on real-world tasks: reaction time, executive function, and perceptuo-motor control.

Reaction time is the latency from the presentation of a stimulus to one or more sensory modalities (e.g., visual, auditory), and a behavioral response (e.g., button press; Welford, 1980; Luce, 1986). Most perceptual and cognitive science experiments measuring reaction times use one of three tasks: simple reaction time, choice reaction time, and serial reaction time tasks (Kosinski, 2010). Simple reaction time tasks involve the presentation of a single stimulus with a single possible response; for example, repeatedly presenting a dot on a computer screen and asking participants to respond as quickly as possible to each dot presentation. Choice reaction time tasks involve the presentation of more than one stimulus, and response types are mapped to each stimulus; for example, presenting a dot or square on a screen and asking a participant to respond by pressing the number 1 or 3 key on a keyboard, respectively, in response to the presentation of each stimulus. Finally, serial reaction time tasks represent a modification of the choice reaction time task, including predictable sequences of each stimulus type. We chose to restrict our analysis to *simple* reaction time as an outcome of interest for three primary reasons. First, simple reaction time resides at a most basic level of cognitive processes, whereas choice and serial reaction time tasks can elicit higher-order processing (i.e., executive function) as evidenced by activation of inhibitory processes and anterior cingulate and prefrontal cortical circuits (Naito et al., 2000; Mulert et al., 2003; Burle et al., 2004). Second, maintaining vigilant attention and quickly reacting to emerging events is critical for sustained performance in a number of high-stakes tasks including those characteristic of military and first-responder operations (Truszczynski et al., 2014; Vrijkotte et al., 2016; Sheffield et al., 2017; Dominski et al., 2018; Brunyé et al., 2020). Finally, reaction time is well-characterized and widely available for efficient download and integration into our modeling approach, and has proven sensitive to variations in acute stress (Lieberman et al., 2002; Olver et al., 2015), sleep loss (Forest and Godbout, 2000; Bartel et al., 2004; Deslandes et al., 2006), and physical exertion (Chang et al., 2012).

Executive function (also referred to as cognitive control) involves developing, flexibly executing, and updating goals and strategies to coordinate performance on relatively complex tasks (Logan, 1985; Miyake et al., 2000). As a higher-order brain function, performing executive functions such as updating goals and inhibiting behavior tends to recruit a very diverse set of cortical and subcortical brain regions including the anterior cingulate, prefrontal cortex, and parietal lobes (Miller and Cohen, 2001; Curtis and D’Esposito, 2003; Seeley et al., 2007; Elton and Gao, 2014). Example executive function tasks typically used in laboratory settings include working memory tasks, the Stroop task, flanker tasks, go/no-go and stop-signal tasks, and problem solving tasks (Donders, 1969; Eriksen and Eriksen, 1974; Rabbitt, 2004; MacLeod, 2005; Lipszyc and Schachar, 2010). We chose to restrict our analysis to executive function tasks eliciting inhibitory control, for two primary reasons. First, inhibitory control is a crucial executive function that has been extensively linked to brain networks and highly relevant performance outcomes such as making shoot/don’t-shoot decisions in law enforcement and military contexts (Biggs et al., 2015; Scribner, 2016; Biggs, 2021). Second, inhibitory control processes are effectively isolated from simple reaction time both behaviorally and neuroanatomically, with largely independent functional brain networks responsible for their performance (Seeley et al., 2007; Elton and Gao, 2014). Finally, inhibitory control has proven sensitive to acute stress (Shields et al., 2016), sleep loss (Drummond et al., 2006; Killgore et al., 2009), and physical exertion (Dietrich, 2006; Dietrich and Audiffren, 2011; Eddy et al., 2015).

Perceptuo-motor control involves the dynamic interaction between sensory and perceptual systems with motor outputs and feedback and is a critical element of adaptive goal-oriented behavior at the level of single effectors (finger, tongue), limbs (arm, leg), and whole-body coordinated movement (Trommershäuser et al., 2003; Verhagen et al., 2008). Examples include dynamically adapting the aim of a weapon relative to a moving target, the swing of a tennis racquet relative to an oncoming ball, or strategically positioning of your feet on a balance beam. Many experiments examining perceptuo-motor control tend to use relatively simple trajectories of single limbs, such as reaching movements, and the control processes involved are thought to maximize goal completion while minimizing biomechanical costs (Sabes and Jordan, 1997; Trommershäuser et al., 2003). From a physiological perspective, controlling body movements involves a dynamic interaction among multiple subcorical and cortical brain regions involved in motor planning and execution (premotor and motor regions), visual perception (somatosensory and occipital regions), and error correction (inferior frontal and cerebellar regions; Gazzola and Keysers, 2009; Tanaka et al., 2009). We chose to include perceptuo-motor control as an outcome variable of interest for two primary reasons. First, perceptuo-motor control underlies myriad tasks in high-stakes contexts including weapons use, driving and aviation, surgical procedures, and firefighting (Pick and Palmer, 1986; Holden et al., 1999; Palmer et al., 2013; Louw et al., 2017). Second, the successful performance of complex perceptuo-motor tasks has proven sensitive to variations in acute stress (Arora et al., 2010b; Bajunaid et al., 2017), sleep loss (Jackson et al., 2013; Connaboy et al., 2020), and physical exertion (Tharion et al., 1997; Grebot et al., 2003).

## Modeling Approach

We developed a model for the conditional probability density function (PDF) of each outcome, here denoted *y*_*i*_, *i* ∈ {1, 2, 3}, given the three predictors, *x*_*j*_ for *j* ∈ {1, 2, 3};^1^ which, by the definition of conditional probability, is computed as

(1) $$\begin{array}{c} p\left(y_{i}|x_{1},x_{2},x_{3}\right)=\frac{p\left(y_{i},x_{1},x_{2},x_{3}\right)}{p\left(x_{1},x_{2},x_{3}\right)}\\ \;=\frac{p\left(y_{i},x_{1},x_{2},x_{3}\right)}{\int p\left(y_{i},x_{1},x_{2},x_{3}\right)\text{d}y_{i}} \end{array}$$

where *p*(*y*_*i*_, *x*_1_, *x*_2_, *x*_3_) is the joint distribution of the *i*-th outcome and the three predictors, *p*(*x*_1_, *x*_2_, *x*_3_) is the joint distribution of the three predictors alone.

We note that the conditional distribution provides a *complete* probabilistic model for the outcome given the three predictors. From this model *any* statistical quantity of interest can be computed including but not limited to mean and standard deviation, mode (the most likely outcome to be sampled from a distribution), median, percentiles, as well as higher order moments such as skew and kurtosis. This characteristic of the model is helpful when users attempt to derive estimates of model confidence, for instance by relying on standard deviation and kurtosis measures.

Constructing the model requires that we determine the *p*(*x*_*i*_), the marginal distributions for the predictors, as well as the joint distribution *p*(*y*_*i*_, *x*_1_, *x*_2_, *x*_3_). As detailed shortly, we also require *p*(*y*_*i*_), the marginals for the outcomes for determining the joint distribution. We compute *p*(*x*_*i*_) and *p*(*y*_*i*_) using density estimation methods applied to data acquired from a variety of original research data sources. Using these estimated marginals as well as effect sizes gleaned from the literature, an optimization-based approach is developed which determines a joint distribution which “best” fits the marginals and the moment information implied by effect sizes. Both processes are detailed below.

### Gathering Model Inputs: Marginal Distributions

For each of the identified predictors and outcomes we estimated PDFs to characterize the marginal distributions. These PDFs serve as inputs into our model. This process involved two primary steps, which we describe in turn.

First, we identified, downloaded, and organized open access data for each of the six variables of interest. To identify extant data, we leveraged longitudinal study databases, primary research reports that included supplementary data alongside the publication and contacted colleagues and authors to request archival data. Table 1 provides a full list of data sources used to define marginal distributions. Table 2 details the primary measures that were identified for each of the six variables of interest, including the number of data points identified. When identifying the primary measures for each variable, we chose to select gold-standard measures that could feasibly be measured and characterized in both laboratory (seated, still) and field (moving, sweating) conditions. For example, when selecting measures of *stress*, we chose to include measures that could be sensed with saliva sensing (i.e., cortisol, alpha amylase), physiological sensing (i.e., heart rate), and subjective stress ratings. In contrast, we chose to omit some specialized measures such as electrodermal activity (EDA), which may be valuable for detecting acute stress states in laboratory settings (Liu and Du, 2018) but relatively limited or challenging to interpret under conditions of thermoregulatory sweating and intense movement (Boucsein et al., 2012; Posada-Quintero et al., 2018). When similar measures (e.g., cortisol) used different measurement units, we converted to a common unit. To calculate error rates for executive function and perceptuo-motor control, we calculated a moving average for each of five successive trials in an original data set.

**TABLE 1:** Original resources for deriving raw data to define marginal distributions (upper row) and effect size estimates to define *xy* relationships (lower row).

Data derived	Original resources
Raw data to define marginal distributions	Brunyé et al., 2013; Giles et al., 2014; Mathiassen et al., 2014; Solis et al., 2015; Inoue et al., 2016; Harris et al., 2017; Hutchinson et al., 2017; Klein et al., 2017; Rosenbaum et al., 2017; Heuberger et al., 2018; Sandra and Otto, 2018; Schumacher et al., 2018; Angelidis et al., 2019; Bock et al., 2019; Goldfarb et al., 2019; Okano et al., 2019; Pyke et al., 2019; Sanabria et al., 2019; Wei et al., 2019; Barrett et al., 2020; Baumert et al., 2020; Fiedler et al., 2020; Holgado et al., 2020; Johnson et al., 2020; Knelange and López-Moliner, 2020; Larsen et al., 2020; Lin H. et al., 2020; Madore et al., 2020; Pahwa et al., 2020; Rodas and Greene, 2020; Rodeback et al., 2020; Timme and Brand, 2020; Tsukahara et al., 2020; Vine et al., 2020; von Helversen and Rieskamp, 2020; Pavlov and Kotchoubey, 2021
Effect size estimates to define *xy* relationships	Lisper and Kjellberg, 1972; Glenville et al., 1978; Larsson, 1989; McMorris and Keen, 1994; Brisswalter et al., 1995, 1997; Hogervorst et al., 1996; Tharion et al., 1997; Forest and Godbout, 2000; Collardeau et al., 2001; Lieberman et al., 2002; Falleti et al., 2003; Grebot et al., 2003; Lee et al., 2003; Moorthy et al., 2003; Bartel et al., 2004; Nilsson et al., 2005; Deslandes et al., 2006; Mouelhi Guizani et al., 2006; Scott et al., 2006; Acheson et al., 2007; Groeger et al., 2008; Arora et al., 2010a; Lim and Dinges, 2010; McMorris et al., 2011; Chang et al., 2012; Jackson et al., 2013; Olver et al., 2015; Shields et al., 2015, 2016; Ludyga et al., 2016; Muley et al., 2016; Starcke and Brand, 2016; Bajunaid et al., 2017; Lowe et al., 2017; Gallicchio et al., 2019; Connaboy et al., 2020

**TABLE 2:** The six variables of interest, including three predictors and three outputs, their primary methods of measurement, and the number of data points used to estimate each variable’s PDF.

Variable type	Variable name	Primary measure(s)	Number of data points	Marginal computation method
Predictor	Stress	Cortisol, alpha amylase, heart rate, and subjective responses	2,066	Mixture modeling + Maximum likelihood
	Sleep	Hours duration	204,403	Kernel density estimate
	Physical exertion	Borg ratings of perceived exertion	4,560	Kernel density estimate
Outcome	Reaction time	Milliseconds latency	20,973	Kernel density estimate
	Executive function	Proportion errors	5,691	Kernel density estimate
	Perceptuo-motor control	Proportion errors	8,265	Kernel density estimate

Second, we estimated PDFs from the data we collected. We used two different methods, depending on whether the data for a variable could be converted to a common unit or not.

#### All Units the Same – Non-parametric Density Estimation

For variables whose datapoints can all be converted to a common unit (sleep, physical exertion, reaction time, executive function, and perceptuo-motor control – see Table 1 for the primary measure for each variable), nonparametric density estimation methods (Izenman, 1991) are employed to construct the PDF. To be concrete, suppose we have *N* data sources for *y*_1_. Then the PDF *p*(*y*_1_) is computed as

$$p\left(y_{1}\right)=\sum_{k=1}^{N}\pi_{k}p\left(y_{1}|F_{k}\right)$$

where *F*_*k*_ denotes the *k*-th data source, π_*k*_ is the fraction of points from the *N* sources coming from *F*_*k*_, and *p*(*y*_1_|*F*_*k*_) is the conditional PDF of *y*_1_ under the conditions associate with *F*_*k*_. This function is computed and manually tuned from the raw data using the kde function from scikit-learn (Pedregosa et al., 2011).

#### Different Units – Mixture Modeling and Maximum Likelihood

For the one variable with multiple primary measures (stress, derived from measures of cortisol, alpha amylase, heart rate, and subjective responses), we needed to map disparate data into a common space. We will illustrate the process in this section. While stress is mentioned here, the process can be applied to any future variable with multiple primary measures.

Assume we have *N* sources of information for stress, *F*_*i*_, *i* = 1, 2, …, *N*. Let *d*_*i*_ be a random variable (in the probabilistic sense) for the data from the *i*-th source. We assume that these data are related to the latent variable stress, *s*, via unknown functions, *g*_*i*_(*s*) which for simplicity in this manuscript we take as affine. Thus, the *j*-th data point from the *i*-th source is

$$d_{i,j}=g_{i}\left(s_{j}\right)=a_{i}s_{i,j}+b_{i}$$

With θ_*i*_ = {*a*_*i*_, *b*_*i*_} the parameters associated with the model for the *i*-th data source, basic properties of derived random variables (Yates and Goodman, 2014) relates the PDF of the observed data given these parameters to the PDF of *s* as

(2) $$p\left(d|\theta_{i}\right)=\frac{1}{\left|a_{i}\right|}p_{S}\left(\frac{d-b_{i}}{a_{i}}\right)$$

In (2) above, the notation $p_{S}\left(\frac{d-b_{i}}{a_{i}}\right)$ indicates the PDF of stress evaluated at the point (*d* − *b*_*i*_)/*a*_*i*_. For *p*_*S*_(*s*), we use a mixture of Gaussians

$$p_{S}\left(s\right)=\sum_{c=1}^{N_{c}}\alpha_{c}g\left(s;\mu_{c},\sigma_{c}^{2}\right)$$

where *g*(*x*; *a*, *b*) denotes a Gaussian PDF in the variable *x* with mean *a* and variance *b* and for *c* = 1, 2, …, *N*_*c*_ we have 0 ≤ α_*c*_ ≤ 1, ∑_*c*_ α_*c*_ = 1, σ_*c*_ > 0, and μ_*c*_ is any real number. In the remainder of this manuscript, we used *N*_*c*_ = 6 with good results. We leave for future work the task of choosing this parameter in an adaptive manner.

Given the data from all *N* sources then, the problem we face is to find the maximum likelihood (ML) estimates of the following parameters:

$$\text{Θ}\equiv \left\{a_{i},b_{i},\alpha_{c},\mu_{c},\sigma_{c}\right\}\text{for}i=1,2,\ldots ,N,c=1,2,\ldots ,N_{c}.$$

To find the ML estimate, we need the joint distribution of the data. Here we assume that all data points are independent draws from their respective *conditional* distributions, *p*(*d*_*i*_|θ_*i*_). We let *N*_*i*_ be the number of observed datapoints from source *i* and *d*_*i*, *j*_ the *k*-th observed datum from source *i* where *j* = 1, 2, …, *N*_*i*_. With *d* the vector of all data from all sources, the joint PDF of the observations is

$$p\left(d|\text{Θ}\right)=\prod_{i=1}^{N}\prod_{k=1}^{N_{i}}p\left(d_{i,k}|\theta_{i}\right)$$

Formally the ML estimate is defined as the solution to the following optimization problem:

(3) $$\hat{\text{Θ}}=\underset{\text{Θ}\in \text{Ω}}{\text{argmin}}\sum_{i=1}^{N}\sum_{k=1}^{N_{i}}-\ln p\left(d_{i,k}|\theta_{i}\right)$$

where Ω accounts for any constraints on the parameters such as the positivity of the σ*_*c*_*. The solution to (3) was obtained using the optimize function in scikit-learn (Pedregosa et al., 2011). The use of this routine requires the specification of an initial estimate for the parameters in Θ. For *b*_*i*_ and *a*_*i*_ we used the sample standard deviation and sample mean of the *i*-th data set. With the *a*_*i*_ and *b*_*i*_ as above, we linearly spaced six Gaussians of identical width and height in the interval [−2, 4]. Concretely, the {α_*c*_, μ_*c*_, σ_*c*_} parameters were initialized to

$$\begin{array}{c} \alpha_{c}=\frac{1}{N_{c}}\sigma_{c}=\frac{1}{N_{c}}\mu_{c}=-2+\left(c-1\right)*\frac{\left(4--2\right)}{N_{c}-1}\\ \;=-2+\frac{6\left(c-1\right)}{N_{c}-1} \end{array}$$

This approach to initialization allows for a natural interpretation of the linear transformation as generalized Z-score coefficients. The initialization process described above treats each data set independently and takes as given the linear transformed data as the basis for fitting the GMM parameters. The maximum likelihood approach requires jointly optimizes *all* parameters across the model allowing for the transformation parameters from one data set to influence those of the other data sets as well as the GMM parameters.

### Gathering Model Inputs: Joint Distributions

For each pairwise combination of predictor and output we estimated correlation coefficients to define their conditional linear relationships. To develop estimates we gathered effect size estimates from original research and meta-analyses. We prioritized meta-analyses when they existed for a particular pairwise relationship, such as stress effects on executive function (Shields et al., 2016).

In some cases, however, meta-analyses did not exist, and we needed to aggregate effect size estimates from a review of original research articles. For example, no meta-analysis exists relating stress and perceptuo-motor control, so we aggregated across original research studies examining this relationship (Arora et al., 2010b; Bajunaid et al., 2017).

Various effect size measures, such as Cohen’s *d* and Hedges *G*, were converted into correlation coefficients (*r*) to derive a common effect size estimate for each of the nine pairwise combinations (3 × 3). The final effect size matrix relating the three predictors and outcomes is detailed in Table 3, and the original sources for deriving effect sizes are detailed in Table 1. Note that a negative-going coefficient indicates a negative association between variables; for example, the negative coefficient relating physical exertion to reaction time indicates that higher physical exertion levels are generally associated with lower (*faster*) reaction times. In contrast, the positive coefficient relating physical exertion with executive function errors indicates that higher physical exertion levels are generally associated with higher (*more error-prone*) executive function task performance.

**TABLE 3:** Effect size estimates (and 95% confidence intervals) relating each of the predictors (stress, sleep, and physical exertion) to each of the outcomes of interest (reaction time, executive function, and perceptuo-motor control).

	Reaction time	Executive function	Perceptuo-motor control
Stress	*r* = 0.220 (0.157, 0.283)	*r* = 0.091 (−0.006, 0.188)	*r* = 0.459 (0.395, 0.523)
Sleep	*r* = −0.278 (−0.363, −0.193)	*r* = −0.183 (−0.251, −0.115)	*r* = −0.189 (−0.334, −0.044)
Physical exertion	*r* = −0.053 (−0.156, 0.05)	*r* = −0.074 (−0.154, 0.006)	*r* = 0.578 (0.223, 0.933)

### Adaptive LASSO Model Optimization for Determining the Joint Density

**FIGURE 1:**
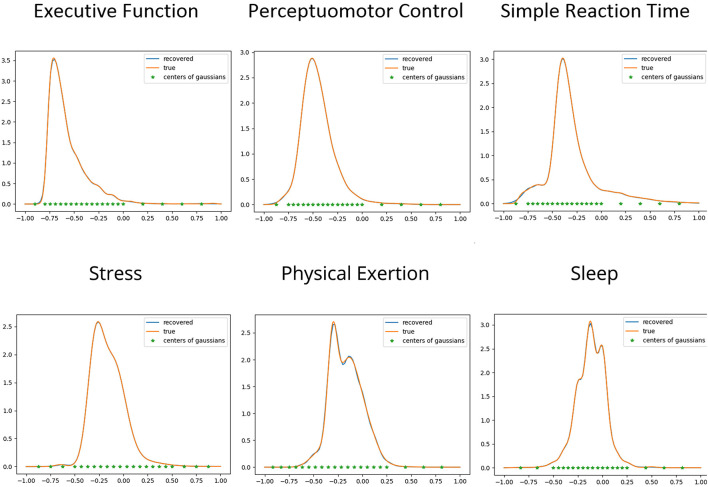
Comparison of true (supplied) and recovered marginal distributions for each of the three dependent **(top)** and independent **(bottom)** variables, along with the centers of the Gaussian kernels, which were computed using the LASSO method detailed in section “Gathering Model Inputs: Marginal Distributions”. Note, the marginals for the dependent variables were generated after separate runs of the fitting algorithm using all three independent variables. The marginal distributions shown for the independent variables were obtained from the fit using reaction time (the marginal fits of the independent variables using the other two dependent variables displayed an equal level of accuracy). In addition to the fidelity between the LASSO model (recovered) and data-generated (supplied) marginals, we also observe that the KDE and ML methods for recovering marginals were able to resolve a variety of detail and non-Gaussian structure within the distributions.

The final component of the model we propose is *p*(*y*_*i*_, *x*_1_, *x*_2_, *x*_3_), the joint distribution of an output variable and all three inputs. In this section, we detail a construction of this PDF as the solution to an optimization problem. Specifically, the four-dimensional PDF is represented as a convex superposition of isotropic Gaussian densities of varying widths uniformly distributed in *z* = (*y*_*i*_, *x*_1_, *x*_2_, *x*_3_). We choose the coefficients in this expansion such that:

• The marginal distributions for *p*(*y*) and *p*(*x*_*i*_) computed using the joint model match in an appropriate sense those constructed from the data as detailed in section “Gathering Model Inputs: Marginal Distributions” above. Figure 1 depicts the supplied and recovered marginal distributions for *p*(*y*) and *p*(*x*_*i*_), also detailed in Table 4.
• The correlation coefficient computed using the joint model similarly, match those obtained from literature in Table 2.
• The LASSO (Tibshirani, 1996) approach resulting in a model which is parsimonious in that only a few coefficients in the expansion are nonzero corresponding to what, in a sense we can make precise, are the “most important” Gaussians.

**TABLE 4:** Supplied and recovered effect size estimates (*r*) for each of the three independent (rows) and dependent (columns) variables.

	Simple reaction time		Perceptuo-motor control		Executive function	
	*Supplied*	*Recovered*	*Supplied*	*Recovered*	*Supplied*	*Recovered*
Stress	0.220	0.216	0.459	0.456	0.091	0.096
Exertion	–0.053	–0.054	0.578	0.576	–0.074	–0.081
Sleep	–0.276	–0.269	–0.189	–0.194	–0.187	–0.187

We chose to employ a LASSO-based approach because of its strong theoretical guarantees. Specifically, the LASSO formulation yields a convex optimization problem to determine the α coefficients. Such problems possess a single, globally optimum solution which can be found regardless of how the parameters are initialized in the search process. This fact along with the robust codes that exist for solving the large-scale instances of such problems as arise in this work make the LASSO alternative quite convenient.

To simplify the notation, in this section, we shall drop the *i* subscript in *y*_*i*_ identifying the specific output variable of interest, as the process is the same for all three outcome variables, and we let *x* = (*x*_1_, *x*_2_, *x*_3_) be the vector of three predictor variables.

While the marginals obtained via the method in section “Gathering Model Inputs: Marginal Distributions” have meaningful units attached to the associated variables, for the purposes of the joint distribution reconstruction we normalize the variable axis of the marginal to fit within the region [−1, 1]. Formally, we specify a lattice of points consisting of *N* equally spaced points (with *N* an odd number) between −1 and 1 on each axis. This will produce a grid of *N*^4^ points in the 4D space. Letting μ_*n*_ ∈ *ℝ*^4^ denote the *n*-th point in this 4D grid, at each of these points we place *M* Gaussians of the form

$$f_{n,m}\left(z\right)=\frac{1}{{\left(2\pi \right)}^{2}\sigma_{m}^{4}}\exp\left(-\frac{1}{2\sigma_{m}^{2}}{||z-\mu_{n}||}_{2}^{2}\right)$$

where the *M* values for σ_*m*_ must be chosen judiciously to allow for a range of “features” in the resulting 4D PDF. For the work here, we found that the following *M* = 7 values were sufficient:

$$\sigma_{m}\in \left\{0.03,0.05,0.10,0.15,0.20,0.30,0.40\right\}.$$

Rather than using two indices, *m* = 1, 2, …, *M* and *n* = 1, 2, …, *N*^4^, we shall use one index *i* = 1, 2, …, *MN*^4^ ≡ *N*_*B*_ so that the above equation now takes the form:

$$f_{i}\left(z\right)=\frac{1}{{\left(2\pi \right)}^{2}\sigma_{i}^{4}}\exp\left(-\frac{1}{2\sigma_{i}^{2}}{||z-\mu_{i}||}_{2}^{2}\right).$$

The underling ordering of the points can be arbitrary, as long as it is used consistently in the software implementation of this method.

Using these Gaussian basis functions, we represent *p*(*y*,***x***)as a mixture model:

(4) $$p\left(y,x\right)=\sum_{i}\alpha_{i}f_{i}\left(y,x\right)$$

where the expansion coefficients satisfy the probability simplex constraints:

(5) $$0\leq \alpha_{i}\leq 1\mathrm{and}\sum_{i=1}^{N_{B}}\alpha_{i}=1.$$

Using this model, the marginals are expressed in terms of the α_*i*_ coefficients as

(6) $$p\left(y\right)=\sum_{i}\alpha_{i}f_{i}\left(y\right)$$

(7) $$p\left(x_{j}\right)=\sum_{i}\alpha_{i}f_{i}\left(x_{j}\right)j=1,2,3$$

Because *f*_*i*_(*z*) is an isotropic Gaussian PDF, *f*_*i*_(*y*) in (6) is a univariate Gaussian with mean equal to the first (that is the “*y*”-th) element of the vector μ_*i*_ and standard deviation σ_*i*_. A similar interpretation holds for *f*_*i*_(*x*_*j*_). To represent the correlation coefficients in terms of the α_*i*_ coefficients, recall that

(8) $$r_{x_{j}y}=\frac{E\left[x_{j}y\right]-E\left[x_{j}\right]E\left[y\right]}{s_{x_{j}}s_{y}}$$

where *s*_*x_j_*_ is the standard deviation of *x*_*j*_ and similarly, for *s*_*y*_. In (8), the means and the standard deviations for the individual random variables are determined from the marginals computed in section “Gathering Model Inputs: Marginal Distributions”. Using (4) in (8) allow us to relate the literature-determined effect sizes to the unknown coefficients as

(9) $$r_{x_{j}y}+\frac{m_{x_{j}}m_{y}}{s_{x_{j}}s_{y}}=\sum_{i}\alpha_{i}\frac{\mu_{x_{j_{i}}}\mu_{y_{i}}}{s_{x_{j}}s_{y}}.$$

To construct a finite dimensional utility function for finding the α coefficients, we uniformly discretize each of the *y* and *x*_*i*_ axes into *K* points. Evaluating (6) and (7) at these *K* points gives 4*K* linear relationships between the α_*i*_ and the marginals. For example

(10) $$p\left(y_{k}\right)=\sum_{i}\alpha_{i}f_{i}\left(y_{k}\right)=A_{1,k}\alpha$$

with *A*_1,*k*_ a row vector containing *f*_*i*_(*y*_*k*_) and α the column vector of the *N*_*B*_ α parameters. Collecting all *K* of these relations gives a matrix-vector model of the form *p*_1_ = *A*_1_α where *p*_1_ is a vector of length *K* and *A*_1_ is a matrix with *K* rows and *N*_*B*_ columns. Similarly, we have *p*_2_ = *A*_2_α, *p*_3_ = *A*_3_α, and *p*_4_ = *A*_4_α for the three sets of constraints of (7). Finally, (9) provides three constraints on the α vector resulting in *p*_5_ = *A*_5_α where *p*_5_ is length three and *A*_5_ has three rows and *N*_*B*_ columns.

A typical LASSO approach for selecting α is known to not provide sparse solutions for problems involving simplex constraints (Li et al., 2020). An iterative procedure overcomes this issue by modifying an adaptive LASSO objective as follows

(11) $$\alpha^{{}^{*}}=\underset{\alpha }{\text{argmin}}\sum_{k=1}^{5}w_{k}{||p_{k}-A_{k}\alpha ||}_{2}^{2}+\lambda {||\text{diag}\left(c\right)\alpha ||}_{1}$$

subject to 0 ≤ α_*i*_ ≤ 1 and $\sum_{i}\alpha_{i}=1$

where ***c*** is a weight vector and diag(*c*) is the diagonal matrix with the vector ***c*** on the diagonal. The approach in Li et al. (2020) solved (11) repeatedly, updating *c* from one iteration to the next to enforce sparsity in α. The formal process is as follows:

(1) Initialize ***c*** to (1, 1, …, 1)^T^
(2) Run the optimization problem with the objective in (11), obtaining a α^∗^
(3) Update the coefficient weight vector ***c*** based on α^∗^:
  $$c_{i}=\left\{ \begin{array}{cc} \frac{1}{\alpha_{i}^{{}^{*}}} & \mathrm{if}\alpha_{i}>t\\ \frac{1}{2\alpha_{i}^{{}^{*}}} & \text{else} \end{array}\right.$$
(4) Threshold α^∗^:
  $\alpha_{i}^{{}^{*}}=0$ if $\alpha_{i}^{{}^{*}}\leq t$, else leave the value unchanged.
(5) Count the number of nonzero elements in the thresholded α^∗^:
  (a) If that number is less than or equal to *N*_*z*_, stop the procedure. The thresholded α^∗^ is the final sparse solution.
  (b) If that number is higher than *N*_*z*_, go back to step 2 using the updated *c* **vector** and repeat the process.

We have found that coefficients with high weights are forced almost to 0, and thresholding these “almost-0” coefficients to 0 has little effect on the recovered joint distribution while significantly increasing the sparsity. A threshold of *t* = 10^−9^ retained this property even on a problem with 1.3 million coefficients. We implemented this procedure in Python using the cvxpy package (Diamond and Boyd, 2016).

In (11), the *w*_*k*_ can be used to weight the different constraints. We found it useful to set a high weight (*w*_5_ = 75) on the *A*_5_ term, which only has three rows, and a low weight (*w*_*i*_ = 1 for *i* = 1, 2, 3, 4) on the other *A*_*i*_ terms, which each have *K* > 3 rows, to balance the importance of fitting the recovered marginal and fitting the recovered *r*-values. The parameter λ is chosen to balance the impact of the first term in the cost function, which encourages a model that fits the marginals and moment constraints well, against the second term which seeks a vector of coefficient that is “sparse;” in that it contains a few large entries with the remainder small or zero. In the context of our iterative procedure, λ affects the rate at which coefficients get pulled to 0 after each iteration: when λ is large, more coefficients get zeroed out. This provides the user with flexibility to target a specific range of coefficients. Larger values of lambda will likely decrease the number of iterations needed. However, this can also lead to overshooting, and the procedure ends up zeroing out all but 10 coefficients (which is usually too sparse). We have found that targeting a representation containing less than 400 coefficients with λ = 1*e*^−5^ to be a decent compromise, but we encourage readers more concerned about time to try larger values of λ.

**FIGURE 2:**
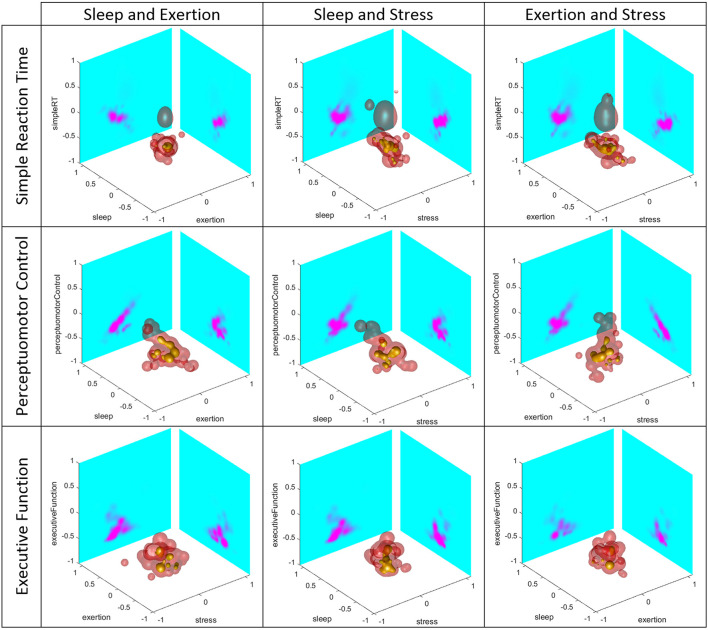
Three-dimensional (3D) slices of the dependent (rows) and pairs of independent (columns) variables. In each panel, we plot the 3D iso-contours at levels 1, 10, and 50% of maximum value in yellow, red, and gray, respectively. The yellow “blobs” indicate the areas of highest probability density in the 3D space, with the red and gray volumes providing an indication as to how the probability is spread. We also display in pink/cyan shades the two dimensional distributions of the dependent and each independent variable with strong pink shades indicating regions of high probability density; these colormaps were saturated at a value of 10% of maximum value to better display the variability in the distribution functions.

For each of the three dependent variables of interest in this study, i.e., simple reaction time, perceptuo-motor control, and executive function, the joint distributions are functions of four variables; namely the single dependent variable and the three independent variables of sleep, stress, and exertion. To visualize these four-dimensional distributions, in Figure 2 we plot 3D slices of the dependent and pairs of independent variables.

As depicted in Figure 2, the marginal plots provide some insight into the way our approach to constructing the 4D distribution functions performs. It is evident that the Adaptive LASSO approach yields high dimensional distributions which, in all cases, faithfully reproduce the six marginals (Table 4). Moreover, the correlation structure of pairs of variables is also evident in the 2D projections; for example, the image on the second row and first column of Figure 2 involving perceptuo-motor control and exertion. The strong diagonal structure seen in the exertion-perceptuo-motor control plane reflects the 0.578 effect size (Table 3) relating these two variables. Analogously, the much smaller effect size of −0.189 between perceptuo-motor and sleep is seen in the other 2D component of this plot, where the directionally is far less pronounced.

## Software Tool Implementation

**FIGURE 3:**
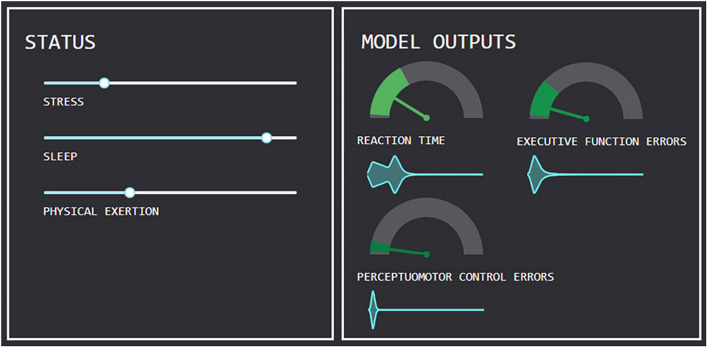
Graphical user interface for a software tool allowing users to interact with computational model, depicting three inputs (*x* variables) and outputs (*y* variables). Shading surrounding output estimates indicates 95% confidence interval around estimated output.

The described model is realized in a set of Python-based software tools that users can rely upon to understand and interact with the model in an intuitive manner. The first iteration of this software tool and graphical user interface is depicted in Figure 3.

The interface provides a means for users to modify each input (*x*) by way of user-inputs (e.g., sliders), and visualize how each predicted output (*y*) is affected. Because the conditional distributions of *y* provide complete probabilistic models for each outcome, it is possible to plot confidence intervals around estimates and visualize outcome distributions. Each of these features is depicted in the software tool, with 95% confidence intervals surrounding each estimated model output, and the distribution visualized as a violin plot alongside each outcome variable.

We envision four primary uses for the model and software tool. First, for scientists and engineers to identify gaps in scientific knowledge and plan research endeavors; for example, when characterizing the relationship between stress (*x*) and perceptuo-motor skill (*y*), we found very few studies experimentally examining this relationship (with a total sample size of only 41 in total), suggesting value in continuing research in this area. As additional predictors and outcomes are integrated into the model, it is likely that many knowledge gaps will become apparent and motivate research agendas.

Second, we envision the model and software tool to be useful for military decision-makers who are planning future operations in the context of limited personnel resources. For example, a commander might preferentially allocate particular units to various tasks that are more or less physically and/or cognitively demanding [commonly termed force management decisions (Wohl, 1981)]. Insights might be made in the context of real-time data feeds from wearable devices and/or may result from commanders’ intuitions about current or projected demands. In addition to force allocation, insights from the tool might be used to prioritize available resources such as food or water and to introduce ample recovery periods (e.g., rest, sleep) following especially intensive activities.

Third, outcomes of our predictive modeling can help prioritize which internal states and contextual factors are worth targeting with environmental sensors (e.g., ambient temperature, noise, and pollution) and wearable biosensors. For example, if mental stress is associated with substantial performance declines across cognitive or physical domains deemed critical for current or projected operations, it may prove particularly advantageous to develop, procure, and/or deploy wearable biosensors to monitor stress states. Recent progress with biosensors placed in the mouth, such as affixed to a tooth, show promise for sensing salivary analytes linked to stress, such as alpha-amylase and cortisol (Robles et al., 2011; Coppedè et al., 2014; Tseng et al., 2018; Ahn et al., 2019).

Finally, outcomes of our predictive model could be used to inform investments into future performance optimization or enhancement techniques and technologies (Bostrom and Sandberg, 2009; Colzato, 2018; Feltman et al., 2019; Brunyé et al., 2020). For example, if physical encumbrance and exertion is particularly influential for sustained performance across domains of interest, it may warrant increased investment in strength and endurance training, lower-extremity exoskeletons, or other approaches for reducing physical and physiological burden of continuous operations (Gregorczyk et al., 2010; Ozaki et al., 2013; Scribbans et al., 2016; Seo et al., 2018; Wei et al., 2020; Shepertycky et al., 2021). The same might be said for sleep or stress, identifying methods for increasing the quality or duration of naps and overnight sleep (Irwin et al., 2008; Ketz et al., 2018; Robinson et al., 2018), and/or reducing the intensity of stress responses (Stanley et al., 2011; Jaremko and Meichenbaum, 2013). As additional predictors are incorporated into the model, it will afford a more robust ranking and prioritization of cognitive and/or physical states that are most negatively impactful for performance and motivate research and development toward mitigating such impacts.

## Limitations and Future Directions

We detailed a preliminary version of a computational model and software tool to enable actionable insights into cognitive and physical performance outcomes by interpreting biosensor data related to sleep status, stress state, and levels of physical workload; given the preliminary nature of the work, there are many directions for continuing research and development.

In Figure 2, we observe distinct spherical structures in these plots in upper left and center right plots. These features arise from our use of isotropic Gaussians of seven fixed widths yielding spherical clusters of similar sizes. Specifically, the LASSO-based scheme chooses a representation of the joint probability density function from a large but finite set of isotropic Gaussians of varying widths on a grid of points in 4D space. The optimization process by which this set is chosen occasionally selects spatially isolated basis functions to help represent small scale features in the marginal distributions. As a result, we see those isolated spherical (since the Gaussian are isotropic) artifacts. To remove these artifacts, our current efforts are aimed at replacing the LASSO scheme with a more flexible method capable of employing Gaussian basis functions with arbitrary center locations and covariance matrices. The cost of such flexibility is an increase in the complexity of the optimization method required to fit the model from data. For example, as the problem is no longer convex in the unknown parameters, we must contend with the challenge of local minima producing poor models. The convex nature of the LASSO scheme used in this manuscript ensures that the model we compute is in fact unique.

The model we developed is ostensibly a main effects model, informed only by effect size coefficients linking single input variables to each output variable. In this manner, no interaction terms are explicitly defined in the preliminary model. However, there could be value in incorporating known interaction terms among our *x* variables, either of the two- or three-way variety. Reproducibility will be important for incorporating interaction terms into the model. In our review of the extant literature relating sleep, stress, and physical exertion to each of our outcome variables, there were very few studies examining interactions among our input variables. Even when those studies did exist, one study might suggest the presence of a two-way interaction between two of our *x* variables when predicting reaction time, and another study might show an opposing pattern or no interaction at all. In studies examining sleep deprivation, physical exertion, and reaction time, some reliable interactions have been found. In one study, 30 h of sleep deprivation caused increased reaction times, but this effect was entirely mitigated by brief bouts of physical exertion (Scott et al., 2006). Two additional studies showed that brief morning exercise routines were sufficient to mitigate reaction time costs of partial sleep deprivation in elite athletes (Taheri and Irandoust, 2020), and that acute submaximal physical exertion counteracted reaction time deficits following a single night of sleep deprivation (Temesi et al., 2013). Continuing model development will begin to incorporate interaction terms to better specify relationships among multiple *x* variables in predicting outcomes. As we pursue the next expanded version of our model, we will be seeking out and incorporating any and all interaction terms that we can identify in the extant literature, and training the model on these patterns. It could be the case that some of these new interactions may qualify or possibly limit the value of the main effects model; it could also be the case that they serve to motivate continuing research to fill knowledge gaps in the literature.

In addition to interaction terms among *x* variables when predicting outcomes, there also likely exist relationships (i.e., collinearity) among both *x* (i.e., *x*-to-*x* relationships) and *y* (i.e., *y*-to-*y* relationships) variables. For example, when individuals are deprived of sleep they also tend to show higher cortisol levels throughout the day and a potentiated HPA axis response to acute stress exposure (Minkel et al., 2014). Sleep loss can also cause bouts of physical exertion to be experienced as more intense than usual, increasing ratings of perceived exertion (Martin and Gaddis, 1981; Myles, 1985) and reducing time to volitional exhaustion (Van Helder and Radomski, 1989). In other words, sleep loss is associated with increased stress levels (Nollet et al., 2020) and altered behavioral responses to exercise. As it is based only on the marginals, construction of the preliminary model does not enforce any specific interactions among the sleep, stress, and physical exertion; however, these patterns will be important to incorporate into continuing model development. The same can be noted for our outcomes of interest; we realize that some *y*-to*-y* relationships may be important to characterize and account for in the construction of the model. For example, slow-downs in reaction time are often accompanied by increases in executive function errors (Willoughby et al., 2020). Populating a complete matrix that expands upon the one detailed in Table 3 will allow us to begin training the model on these *x*-to*-x* and *y*-to-*y* relationships.

Incorporation of the interactions described in the previous two paragraphs into our probabilistic models can easily be accomplished depending on the nature of the data provided. For example, from jointly collected observations of two input variables, say *x*_1_, and *x*_2_ along with one output *y*, density estimation methods can produce the corresponding PDF *p*(*y*, *x*_1_, *x*_2_) which will provide another *A* matrix to be fit in the LASSO process. Analogous methods would be used if we were provided data from multiple output and inputs or just multiple inputs.

Whereas time is an inherent property of our sleep variable, it is not incorporated into any other aspect of the model. However, we also know that the influence of some *x* variables will be time-dependent. For example, extended bouts of moderate- to high-intensity physical exertion are likely more impactful than relatively brief bouts. Indeed meta-analytic investigations of the relationship between physical exertion, duration, and cognitive performance indicate that 1–10 min of exercise carries an effect size of 0.06 with cognitive task performance, whereas 11–20 min shows an effect size of −0.18, and greater than 20 min has an effect size of 0.26 (Chang et al., 2012). Notice how the effect sizes characterizing the relationship between physical exertion and cognition change in magnitude and directionality. In other words, the relationship between physical exertion and cognitive performance may interact with duration in a non-linear manner. There are at least two ways for the model to incorporate such patterns. First, we could develop a new predictor that combines physical exertion and duration into a single exposure variable that replaces the original physical exertion variable; this approach would be dependent on original data sources to help characterize the relationship between exertion- and duration-contingent effects on cognitive outcomes. Second, we could develop multiple marginal input distributions for physical exertion, one each for relatively brief, moderate, and long-duration periods of exertion. Similar approaches may need to be investigated for extended durations of stress, which may shift stress responses from acute to chronic and alter the relationship with our outcomes of interest.

The three predictors and outcomes of interest are by no means an exhaustive representation of the myriad individual (e.g., traits, skills, and experience) and contextual (e.g., thermal load, hydration, and group dynamics) factors, and cognitive and physical performance outcomes characterizing real-world performance. As we continue to develop the model, we will begin to incorporate additional predictors and outcomes that help describe the individuals in a sub-population, and more comprehensively describe predicted outcomes. For outcomes, near-term goals are to incorporate three additional outcomes of interest: gross motor ability (e.g., strength, endurance), memory (e.g., recognition, recall), and communications (e.g., verbal language production and comprehension).

While the present model and tool are designed to ingest and interpret data from wearable devices, either in real-time or with opportunistic sampling, we have not yet integrated sensing and interpretation. With some input variables, such as sleep, there are near-term opportunities for such integration. For example, wearable sleep trackers are becoming common on the consumer market, with options providing daily tracking of sleep quantity and quality. Using either discrete manual updates by users or integrating with a device’s application programming interface (API), the model could receive daily updates to feed the sleep input parameter. Over the next several months, we will be exploring integration of sensor outputs into our model, beginning with sleep and continuing to incorporate physical exertion and stress sensor feeds. While this research topic is specifically related to wearable sensing technologies, it should also be pointed out that many non-contact in-situ sensing technologies are emerging on the market. For example, non-contact stress detection is possible through infrared and microwave doppler radar (Zakrzewski et al., 2012; Lee et al., 2015; Shan et al., 2020), and doppler radar may be used to non-contact measurements of the orientation (and possibly quality) of sleep (Kiriazi et al., 2021). It is possible that non-contact biosensors may meet or exceed the sensitivity and reliability of wearable sensors in the future, may prove valuable complements to wearable sensors, and may increase the breadth and specificity of measurements that could be used in predictive models. One advantage of our approach to modeling several input data types is that we are relatively sensor-agnostic, which ultimately should increase the flexibility of our model to accept novel non-contact sensor inputs.

The current version of the model is trained on both laboratory and field study data, but model predictions have not yet been validated in either setting. We are currently collecting both laboratory and field data in studies intentionally designed to manipulate and/or measure sleep, physical exertion, and mental stress. In these studies, we are measuring a diverse number of cognitive and physical task outcomes that are directly applicable to the output variables of our model. Two specific studies are worth detailing. First, we are conducting an immersive virtual reality study that elicits high levels of mental stress via threat of aversive torso shock (versus a low stress control condition). In this study, we measure performance outcomes including reaction time, recognition memory, executive function, decision making, and perceptuo-motor control (marksmanship aiming). Outcomes from this first study will help validate predictions linking mental stress to the three model outputs, while also providing helpful data to begin expanding the breadth model predictions. Second, we are conducting an exercise physiology study that elicits high levels of physical exertion via a prolonged (2-hour) inclined treadmill walk while carrying a heavy backpack load (versus a no-load control condition). In this study, we measure performance outcomes including reaction time, recognition memory, executive function, decision making, and gross motor output. Outcomes from this second study will help validate predictions linking physical exertion to the three model outputs, additionally providing preliminary data to expand the breadth of model predictions. Due to the SARS-CoV-2 pandemic and associated human subjects research restrictions, these studies have been delayed; we anticipate completing these studies and model validation over the next year. As we continue to collect data in these studies, we will use a portion of these data to continue populating and expanding our model, and a portion to validate model predictions. Based on the fit of model predictions to our data, we will iteratively expand upon training data and/or continue to increase the breadth of the model inputs and outputs.

Finally, the current version of the model and software tool does not fully bridge the gap between our predictors of interest (stress, sleep, and physical exertion) and actionable intelligence for military applications. Whereas scientists and engineers may be interested in predicting such aspects of performance as reaction time and executive function errors, and some trainers and commanders may understand and realize how to use these predictions in their own planning and operations, these specific processes and measures may not be comprehensible or useful for all users. However, we argue that any outcome of interest in an applied setting, such as marksmanship or threat detection ability, can be summarized from weighted combinations of model outputs. For example, task analyses of marksmanship ability demonstrate that aspects of gross and fine motor ability, perceptuo-motor control, and executive function are involved (Chung et al., 2004). Weighted combinations of these output variables can give rise to indirect predictions of marksmanship ability through an understanding of its component processes. This methodology will likely prove valuable when translating model predictions to user-specific needs across a range of applications.

## Conclusion

As wearable sensors continue to proliferate the consumer market, they provide opportunities to derive quantitative measures and inform actionable insights into predicted performance outcomes across cognitive and physical domains (Lin et al., 2020). Communicating information to users from wearable devices is the first step in realizing this opportunity, with the second step involving the interpretation and translation of data into predicted outcomes. These predicted outcomes can be used to help plan continuing operations, allocate groups of individuals to tasks with specific demands, identify gaps in knowledge and opportunities for biosensing, and inform enhancement strategies to help mitigate performance-deleterious effects of *x* variables.

As a first step toward addressing this challenge in the cognitive and physical domain, we detailed an extensible computational model that can translate inputs from wearable actigraphy/sleep sensors, inertial measurement units (IMUs), and/or physiological stress biosensors to actionable insights for leaders and trainers. While preliminary in nature, our model provides a strong foundation for continuing development of human performance predictive capabilities in the cognitive and physical domains.

## Data Availability Statement

The original contributions presented in the study are included in the article/supplementary material, further inquiries can be directed to the corresponding author.

## Author Contributions

TB, EM, and KY drafted the manuscript and revised and expanded the manuscript upon by all other authors. All authors contributed to the article and approved the submitted version.

## Conflict of Interest

The authors declare that the research was conducted in the absence of any commercial or financial relationships that could be construed as a potential conflict of interest.

## Publisher’s Note

All claims expressed in this article are solely those of the authors and do not necessarily represent those of their affiliated organizations, or those of the publisher, the editors and the reviewers. Any product that may be evaluated in this article, or claim that may be made by its manufacturer, is not guaranteed or endorsed by the publisher.

## Footnotes

1 For the remainder of this manuscript, we let *y*_1_ = reaction time, *y*_2_ = executive function, *y*_3_ = perceptuo − motor control, *x*_1_ = stress, *x*_2_ = sleep, and *x*_3_ = physical exertion.
